# An evaluation framework for operational interventions on urban mass public transport during a pandemic

**DOI:** 10.1038/s41598-023-31892-2

**Published:** 2023-03-30

**Authors:** Ramandeep Singh, Daniel Hörcher, Daniel J. Graham

**Affiliations:** Transport Strategy Centre, Centre for Transport Studies, Department of Civil and Environmental Engineering, Imperial College London, Exhibition Road, London, SW73AE UK

**Keywords:** Civil engineering, Applied mathematics

## Abstract

Decision making in a rapidly changing context, such as the development and progression of a pandemic, requires a dynamic assessment of multiple variable and competing factors. Seemingly beneficial courses of action can rapidly fail to deliver a positive outcome as the context changes. In this paper, we present a flexible data-driven agent-based simulation framework that considers multiple outcome criteria to increase opportunities for safe mobility and economic interactions on urban transit networks while reducing the potential for Covid-19 contagion in a dynamic setting. Using a case study of the Victoria line on the London Underground, we model a number of operational interventions with varied demand levels and social distancing constraints including: alterations to train headways, dwell times, signalling schemes, and train paths. Our model demonstrates that substantial performance gains ranging from 12.3–195.7% can be achieved in metro service provision when comparing the best performing operational scheme and headway with those realised on the Victoria line during the pandemic.

## Introduction

Public transport plays a key role in the everyday functioning of large dense cities, and it supports the operation and productivity of local and national economies. Under normal circumstances, mass shared use is the key characteristic that renders public transport sustainable and economically efficient, but amid the threat of an infectious disease, the trade-off between disease containment and the desire for mobility presents huge challenges. Furthermore, the rapidly changing conditions during a pandemic requires consideration of multiple variable and competing factors in a dynamic setting. In this paper, we present a flexible data-driven agent-based simulation platform that evaluates multiple outcome criteria to increase safe travel and economic interactions on urban transit networks while reducing the potential for contagion. We note the while our work is motivated by the particular experience of Covid-19, it provides insights that generalise to any context that requires restrictions on social interactions and mobility, including future pandemics.

SARS-CoV-2, the virus pathogen associated with the Covid-19 disease, is thought to be transmissible directly via droplets or aerosols and indirectly via fomite tranmission^1,2^. Due to the physical proximity of people in enclosed spaces, public transport networks are considered potential environments where virus transmission can take place intensely. Indeed, there are a number of empirical and theoretical studies demonstrating the potential for SARS-CoV-2 to spread within public transport networks (refer to a review^3^ of relevant papers). Moreover, many studies report positive associations between mobility in general and the incidence of Covid-19 cases due to mixing of populations between different locations^4–8^.

In response, most governments around the world have imposed restrictions on travel to inhibit transmission of the virus. The mechanisms of virus transmission, travel restrictions, and subsequent impacts on perceptions of travel during the pandemic have led to markedly reduced demand levels on public transit networks^9–16^. For rail modes in particular, the Transport Strategy Centre (TSC) at Imperial College London have been tracking demand changes over the pandemic for over 40 metro operators via the COMET metro benchmarking group^17^. The TSC report that average demand levels dropped to below 40% of pre-pandemic levels across all metros after the initial lockdowns in early 2020, with metros in Europe and North America experiencing more severe demand reductions with average levels reaching approximately 10% of pre-pandemic demand. As of the last quarter of 2021, demand has not yet fully recovered, with levels ranging from 40–75% of pre-pandemic levels across all metros in the COMET group.

Though there are vast numbers of studies reporting the extent to which public transport demand has reduced, research and implementation of modelling platforms to test best practice transit operations in the context of reduced demand levels is limited. Moreover, though some cities around the world have begun relaxing social distancing requirements, many other cities have kept the requirement, particularly in response to the spread of the most recent Omicron variant, and so operators must continue to consider the need to accommodate social distancing policies in their operations. Under regular conditions, transit operators typically target full utilisation of vehicles up to their maximum physical capacities. Under pandemic conditions, however, operations must be adjusted to accommodate lower levels of demand and social distancing requirements. Though many transit operators possess in-house network models, they are typically calibrated to a narrow set of operating conditions and are often too inflexible and intractable to model the atypical demand and supply conditions imposed during the pandemic. Furthermore, these applied models are usually not informed by the latest evidence from the academic literature, which we believe provides a solid empirical foundation for simulation.

This research creates a flexible and tractable agent-based simulation platform to test the effectiveness of operational interventions on urban mass transit during the Covid-19 pandemic. The model comprises three main parts: (i) definition of train and passenger movements, (ii) allocation of individual agents (or passengers), and (iii) multidimensional performance evaluation. The operational interventions modelled include: alterations to headways, dwell times, signalling schemes, and alterations to train paths. We use the simulation model to undertake a case study of the Victoria line on the London Underground using open-source data from Transport for London (TfL).

The paper contributes to the literature from a number of perspectives. To our knowledge, only three studies^18–20^ present a quantitative framework to evaluate demand and/or supply of transit networks under pandemic conditions. The objective criteria is either to minimise operator and passenger costs^18^, or to minimise Covid-19 infection levels^19,20^. These approaches assume that (i) demand is unresponsive to exogenous supply-side interventions and (ii) do not include an evaluation of the societal economic benefits of travel. In our model, we explicitly specify that passenger demand is responsive to supply, and this is a key contribution of our work. In the multidimensional performance evaluation module, we allocate all passengers in an unconstrained queue and assign heterogeneous and correlated values to specify the willingness to pay and value of time of each passenger. Passengers travel if their willingness to pay is greater than their generalised cost; if not, passengers are removed from the queue, and the next passenger in the queue moves ahead thus updating their generalised cost. The model is re-run in an iterative manner until stabilisation i.e. when all passengers who opt not to travel are removed. Furthermore, we adopt a multidimensional performance criterion which captures the trade-off between costs incurred by operators and the economic benefits of travel at a societal level.

The train movement module of our model is based on cellular automata (CA) principles. The concept of CA modelling was first applied in transport applications by^21^ for road traffic, and was later built upon by^22^ who introduced the seminal Nagel-Schreckenberg model of road traffic flows. CA has since been adapted for rail applications^23–25^. In our model, we extend beyond the conventional modelling of train movements only, and additionally incorporate passenger flows to enable calculation of individual passenger itineraries. Moreover, our model is flexible such that a wider range of operational interventions beyond those tested earlier^18–20^ can be generated. The flexibility of the model also enables the incorporation of up-to-date empirical estimates of operational characteristics and passenger travel behaviour. In this application of the model, we include the most recent empirical estimates on metro operational costs including returns to density effects^26^. The results from the simulation framework enable the relative disadvantages and benefits of different operational and demand schemes to be quantified. These outputs can in turn be used by transit authorities and operators to make evidence-based decisions on interventions for travel demand and capacity management during pandemic conditions.

## Methods

**Figure 1:**
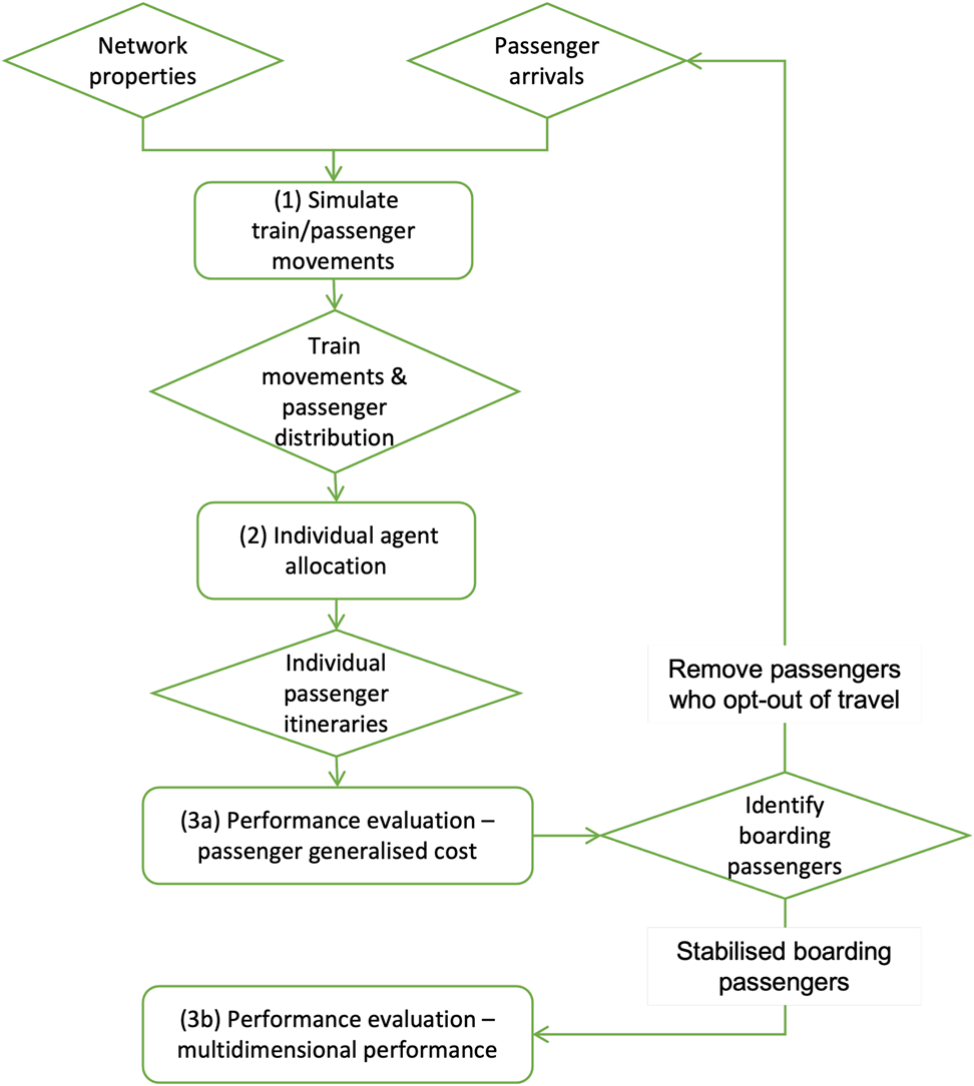
Summary of modelling process.

As shown in Figure 1, the model comprises three main components: (1) the definition of train and passenger movements, (2) the allocation of individual agents (passengers), and (3) the performance evaluation (split into 3a and b in the figure). In (1), the train and passenger movements are simulated and outputs are generated on the position and speeds of each train and the distribution of passengers on each train and at each station at every time step. In (2), each passenger is allocated to a train on a first-come-first-serve basis and individual passenger itineraries are defined. In (3), the generalised cost of travel for each passenger is calculated and it is determined whether each passenger makes the decision to travel or not. The passengers who opt out of travelling are removed, and (1), (2), and (3a) are re-run until stabilisation is reached where all passenger who chose not to travel have been removed. Final calculations of the multidimensional performance criterion (3b) are then undertaken with the stabilised set of passengers who opt to travel.

In the following section, we summarise the main components of the model. Further details on explicit train and passenger movement rules are presented in the Supplementary Material in Appendix A. All modelling is undertaken using R statistical analysis software.

### Train and passenger movements

As mentioned, we adopt the CA modelling framework to define train movements. Unlike conventional models which stop at the modelling of train movements only, we extend the model to incorporate passenger flows. This enables individual passenger itineraries to be generated. We model at the level of individual lines, and define a one-dimensional space as discrete cells enabling movement in one direction. Each cell is allocated a given state i.e. empty/occupied. The state of each cell at time $t+1$ is determined by rules based on the current cell’s state at time *t* and the state of surrounding cells. The following train movement simulation framework corresponds to a moving block signalling system.

The governing rules for train movement are as follows:

(i) Trains travel at their maximum specified velocity, unless boundary conditions impose constraints.
(ii) Trains must maintain a minimum separation distance $\delta$ from the end of the previous train to the front of the current train as follows:
  1 $$\begin{aligned} \delta =d_{b}+d_{s} \end{aligned}$$
  where $d_{b}$ is the braking distance as per the fundamental equation of motion:
  2 $$\begin{aligned} d_{b}= \frac{v^2}{2b} \end{aligned}$$
  where *v* is velocity of train and *b* is the deceleration/braking capacity of the train. $d_{s}$ is a fixed safety distance.
(iii) Trains stop at stations to enable passenger boarding/alighting. The dwell times are calculated when the train reaches the station platform for the first time. A degree of randomness is introduced to reflect realistic dwell time movements.
(iv) New trains are introduced at the specified headway value, subject to minimum train distance constraints. A degree of randomness is incorporated into the headway value to reflect realistic train dispatch.

Passenger movements are integrated within the train movement model. From the initialisation of the simulation time, passenger arrivals begin at station platforms. Passengers are assumed to follow a random Poisson arrival process as is conventional for high frequency transit networks (refer to the authors' previous work^27^ and references therein). When trains stop at stations, passengers board and alight during the specified dwell time subject to train capacity and boarding rate constraints.

### Individual agent allocation

From the train movement modelling output of passenger volumes at each platform and on-trains, we assign journey itineraries to each individual passenger. For each passenger *i*, we calculate the following quantities: passenger arrival time $y_{io}^{\text {entry}}$ at the origin station *o*, passenger boarding time $y_{ino}^{\text {board}}$ on train *n*, passenger alighting time $y_{ind}^{\text {alight}}$ at the destination station *d*. It should be noted that we do not specify station entry times at the origin station nor station exit times at the destination station; each passenger itinerary represents passenger wait times and on-train times only. The general process of allocating passengers to unique itineraries is as follows:

1. From the simulation modelling output we calculate total passenger arrivals $Q_{\gamma sn}$, boards $Q_{\beta sn}$, and alights $Q_{\alpha sn}$ for every station *s* per train *n* over the total simulation time. Missed boards are calculated as $Q_{\varepsilon sn}=Q_{\gamma sn}-Q_{\beta sn}$. The missed boarding passengers are allowed to queue at the station platform in an unconstrained manner.
2. We then calculate origin-destination flows for every train using iterative proportional fitting (IPF) to ensure that the row sums i.e. $Q_{\beta sn}$ and column sums i.e. $Q_{\alpha sn}$ are achieved. The ‘ipfr’ package in R is used to perform the IPF. Since the OD flows obtained from the IPF result in decimal values, we apply an integerisation method^28^ to ensure that OD flows are integers representing whole passengers.
3. From OD flows per train, we randomly allocate destination stations to each passenger, and allocate arrival times at the origin station according to the known Poisson arrival process of passengers at each station.
4. Passengers are then allocated to trains on a first-come-first served basis, and wait $y_{i}^{\text {wait}}$ and in-vehicle $y_{i}^{\text {ivt}}$ times are calculated as follows. (Note: all journey times are measured in seconds).
  3 $$\begin{aligned}{} & {} y_{i}^{\text {wait}}=y_{ino}^{\text {board}} - y_{io}^{\text {entry}} \end{aligned}$$
  4 $$\begin{aligned}{} & {} y_{i}^{\text {ivt}}= y_{ind}^{\text {alight}} - y_{ino}^{\text {board}} \end{aligned}$$

### Performance evaluation

Our multidimensional performance criterion includes the net economic benefit of travelling to users alongside operator costs. The agent-based representation of travel demand enables us to introduce user heterogeneity with high granularity, allowing synthetic travellers to have unique but correlated trip benefits and travel time valuations. This approach is novel in the context of large-scale agent-based transport simulators.

First, the generalised cost of travel, that is, the sum of monetary and non-monetary costs, is calculated for each passenger. The modelling platform enables the value of time and willingness to pay parameters to be specified as being heterogeneous across passengers; they can be allocated as correlated (or uncorrelated) distributions with appropriate forms such as log-normal or uniform distributions. The generalised cost of travel of each passenger $gc_{i}$ is as follows:

5 $$\begin{aligned} gc_{i}= tp + vot \cdot y_{i}^{\text {ivt}} + wt \cdot vot \cdot y_{i}^{\text {wait}} + cf_{p} \cdot vot \cdot y_{i}^{\text {wait}} + cf_{t} \cdot vot \cdot y_{i}^{\text {ivt}} \end{aligned}$$

where *tp* is the ticket price, *wt* is the wait time multiplier, and $cf_p$ and $cf_t$ are crowding multipliers for platform and train crowding, respectively.

A passenger is considered to travel if their willingness to pay is greater than their generalised cost, i.e. if $wtp_{i} > gc_{i}, i \in \theta$ where $wtp_{i}$ is the willingness to pay of passenger *i*, $gc_{i}$ is as previously defined, and $\theta$ is the set of passengers that take the decision to travel. Those passengers who opt not to travel, i.e. in cases where $wtp_{i} < gc_{i}$, are removed from the arrivals process. This then enables the next passenger in the queue to move ahead and be considered to board, thus updating the wait times and in-vehicle times (if a previously queuing passenger boards) of all passengers in the queue. The calculations are re-run in this manner in an iterative process until stabilisation, i.e. until all passengers who do not opt to travel are removed.

We are then able to compute the consumer surplus $cs_{i}$ for each passenger *i* that decides to travel. The consumer surplus represents the economic benefit accrued by each individual user for making the decision to travel, and it is defined as:

6 $$\begin{aligned} cs_{i} = wtp_{i} - gc_{i} \end{aligned}$$

To assess the viability of the operational scheme, we adopt a multidimensional performance criterion *MDP* which is analogous to the concept of social welfare in transport economic theory; the criterion captures multiple and often conflicting elements including net user economic benefits of travel through the quantification of total consumer surplus over all travelling passengers, operator revenue from ticket sales, and operator costs. *MDP* is defined as:

7 $$\begin{aligned} MDP = CS + MCPF \cdot PR \end{aligned}$$

where *MCPF* is the marginal cost of public funds, *CS* is the total consumer surplus over all travelling passengers $n_\theta$:

8 $$\begin{aligned} CS= \sum _{i} cs_i, i=1,..., n_\theta \in \theta \end{aligned}$$

and *PR* is the profit, calculated as

9 $$\begin{aligned} PR= (tp \cdot n_\theta ) - \left( (\kappa * n_{\text {cars}})^{RTD}*\phi \right) \end{aligned}$$

where $\kappa$ is the total cumulative number of kilometres travelled by all trains over the simulation time period, $n_{\text {cars}}$ is the number of cars per train. *RTD* is the average returns to density scaling factor which reflects the relative impact of intensity of use on cost^26^, and $\phi$ is the average cost per car km. The best performing operational scheme is that which takes the highest value of the multidimensional performance criterion *MDP*.

## Case study - Victoria line, London underground

### Network, rollingstock, and passenger inputs

We model the Victoria line on the London Underground metro system, and use rollingstock, line-layout, and passenger movement parameters from open-source data provided by TfL^29,30^. We make assumptions on the maximum train velocity, acceleration and braking rates, and the required safety distance between trains, with parameter values set to previous estimates^31^. The maximum passenger movement rate (i.e. boards and alights) through all doors for a standard 8 car train is set as $q_{\text {max}}=29$ passengers/second^32^. The station positions along the line correspond to all 16 stations from Brixton to Walthamstow Central on the Victoria line in the northbound direction.

### Performance evaluation inputs

As specified in a previous publication^33^, we assume that the value of time and the willingness to pay for each passenger are correlated according to a multivariate normal distributional form with a correlation coefficient of 0.50. We make the assumption that the value of time is log-normally distributed with a mean of $\$$20/h and standard deviation of $\$10$/h, and the willingness to pay is uniformly distributed between $\$0$ and $\$100$ as per^33^. The wait time multiplier *wt* is taken as 1.5 as per TfL standards for metro journey times^34^, and the ticket price *tp* is set to $\$2$ per ticket.

For platform crowding, we calculate the average passenger density on the platform between train arrivals $p_{p}$ (passengers per m$^2$), and use this quantity to calculate the platform crowding multiplier in accordance with TfL standards^34^ as per Eq. (10). For train crowding, we calculate the average number of passengers per train $p_{t}$ and calculate the train crowding multiplier in accordance with TfL standards^34^ as per Eqs. (11 and 12). As shown in Eq. (12), we make an amendment to the TfL guidelines; if we find that the multiplier $\Lambda <1$, then we discount its application as this implies that passengers derive a benefit from lower levels of crowding. However, as we model pandemic conditions with imposed upper limits on train capacity for social distancing, we do not believe that passengers would perceive this to be an added benefit of travel, rather, passengers would expect lower levels of crowding to be mandated as a baseline.

10 $$\begin{aligned}{} & {} cf_p = {\left\{ \begin{array}{ll} 0 &{} \text {if} p_p < 0.5 \\ 4.0 &{} \text {if} p_p > 2\\ 2.5 + (0.667*(p_p-0.5)^2) &{} \text {if} 0.5 \le p_p \le 2 \\ \end{array}\right. } \end{aligned}$$

11 $$\begin{aligned}{} & {} \Lambda = 0.09 + 1.823\left( \frac{p_{t}-280}{748}\right) \end{aligned}$$

12 $$\begin{aligned}{} & {} cf_t = {\left\{ \begin{array}{ll} 0 &{} \text {if} \Lambda < 1 \\ \Lambda &{} \text {if} \Lambda \ge 1 \\ \end{array}\right. } \end{aligned}$$

It is difficult to obtain estimates of the impact that the Covid-19 pandemic has had on passenger perceptions of crowding as the severity of the pandemic has been regularly fluctuating and thus perceptions are subject to change quickly. However, to account for potential additional passenger sensitivity to crowding during Covid-19, along with modelling baseline crowding perceptions, we undertake sensitivity analysis of crowding perceptions by trialling the application of additional multipliers to the platform crowding multiplier $cf_p$ and train crowding multiplier $cf_t$. The multipliers trialled are 0 (no crowding effects), 2, and 5.

In the final calculation of the multidimensional performance criterion, the marginal cost of public funds *MCPF* is taken as 1.2^33^.

### Demand and operational scenarios

We trial 4 demand levels and 2 maximum train capacity levels. The demand levels are as follows: 35%, which corresponds to the average demand level on the London Underground throughout the 2020 pandemic period, 50%, 75% and 100% of 2019 weekday demand levels for the Victoria line^30^. The base demand data levels (i.e. 100%) correspond to demand levels during weekday PM peak periods in 2019 on the Victoria line. The maximum train capacities trialled are: $M=180$ passengers which corresponds to 4m$^2$ social distancing for standing passengers (2 m straight line distance) and every second seat occupied; and $M=290$ passengers which corresponds to 1 m$^2$ social distancing for standing passengers (1 m straight line distance) and every second seat occupied.

We trial 4 different operational schemes over headways ranging from $h=60$ to $h=240$ s:

1. Moving block operations - This is the base operational condition trialled. The minimum dwell time to enable alighting movements $C_{\alpha }$ is set as 15 s in line with minimum dwell times on the Victoria Line.
2. Moving block + 25 s and 35 s minimum alighting time - In these two options we wish to investigate the effect of increasing minimum dwell times for alighting movements and thus set $C_{\alpha }$ to 25 s and 35 s. Increasing dwell times may yield better performance in higher demand and higher capacity conditions as trains are able to load more passengers at each stop.
3. Fixed block operations - Though the Victoria line and many newer metro lines around the world operate via a moving block signalling scheme, many older lines operate using the fixed block system. Under this scheme, the line is divided into ‘blocks’ and each block may only be occupied by one train at any given time. We trial equal block lengths of 1200 m, which conservatively accommodates two trains and separation distances. The fixed block system may perform worse than the moving block system, particularly at shorter headways, as the required separation distances between trains are likely to be longer.
4. Skip-stop operations - Trains stop at every second station in an alternating fashion. Approximately 47% of passengers require a transfer to travel from even to odd stops (and vice versa) and we assume that this occurs at the trip end; we assume that the passenger alights at one stop after the desired destination, and turns back making use of the opposite (southbound) direction. We assume that passengers would be opposed to two interchanges in one trip, and so we specify that all train runs stop at the terminal stop in the line. An additional fixed penalty of 3.5 min transfer time is applied in the calculation of the generalised cost in Eq. (5) in line with TfL specifications^34^.

Prior to the pandemic, the regular operating headway of the Victoria line in 2019 during weekday PM periods was 100 s. As the Victoria line runs through central London, minimal restrictions were imposed during the pandemic as the line was still required to serve essential workers. Two stations, Blackhorse Road and Pimlico, were closed entirely from 21 March 2020 to 18 May 2020^35^. We are not aware of any headway changes for the weekday PM peak period during the pandemic. After 18 May 2020, the Victoria line resumed regular operations with no additional changes; all stations were operational and weekday PM peak hour headways were 100 s.

For the two month period from 21 March 2020 to 18 May 2020 when the two stations were closed, demand on the London Underground was 5% of 2019 levels^36^. Since we do not model the scenario where demand is at 5%, and since the Victoria line predominately ran regular operations of 100 s with all stations operational for the majority of the pandemic, we will assume that the base case of operations to compare against during the pandemic is a 100 s headway with all stations operational using the base moving block scheme.

## Results and discussion

Comparing across all operational schemes, the skip-stop scheme performs worst in all cases. In a majority of cases, the multidimensional performance criterion yields a negative value of performance for the skip-stop scheme, while all other schemes generate positive values in all cases. The substantially lower level of performance is likely due to the fact that the skip-stop scheme leads to much longer wait times and the need for some passengers to undertake interchange movements, which greatly increases the generalised cost of trips. Due to its substantial relative under-performance, we exclude further discussion of the results of the skip-stop scheme for the remainder of this section.

**Figure 2:**
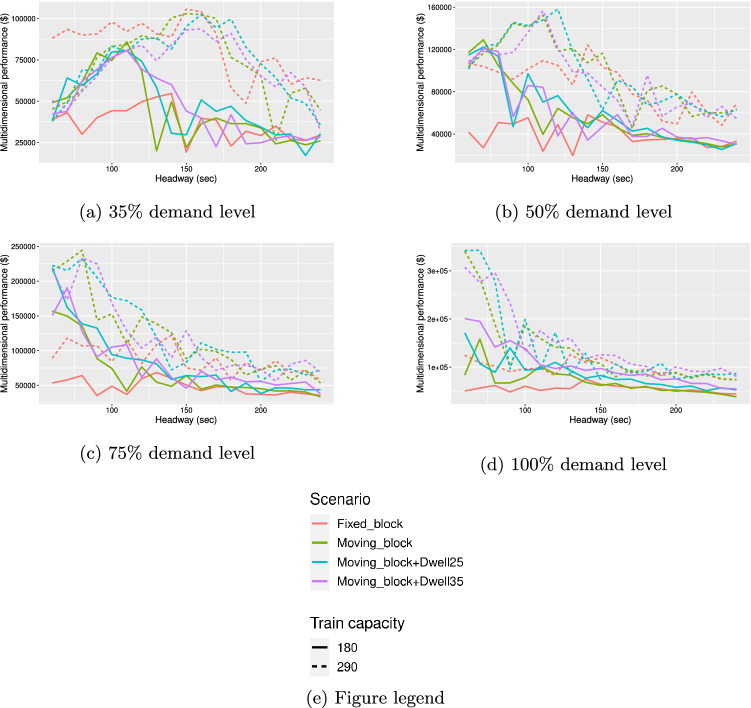
Performance vs headway for all operational scenarios, baseline Covid-19 crowding multiplier of 1.

For the remaining operational schemes, i.e., the base moving block, the moving block with 25 s minimum dwell time, the moving block with 35 s minimum dwell time, and the fixed block scheme, the results for the multidimensional performance values vs headway are illustrated in Fig. 2. It is worth noting that the random fluctuations in the graphs is due to the stochastic allocation of individual passenger value of time and willingness to pay values for each iteration of the models.

### Best performing headway

In terms of general findings, there is a trend that at lower levels of demand, best levels of performance are attained when fewer trains are run at longer headways to meet the low demand levels. As demand increases, the best performing value of headway reduces. This finding is in line with the theoretical literature of public transport provision; the best performing value of headway decreases with increasing demand, as pronounced waiting time and crowding cost savings justify the operating cost of running more frequent trains. Furthermore, for a given level of demand, there is a general trend that the best performing value of headway is longer for the 1m social distancing scenario compared to the 2 m social distancing scenario. This result is as expected and plausible; at a 1 m level of social distancing, each train is able to carry more passengers, and thus fewer trains are required to serve passenger demand. Moreover, the best performance level is higher under more relaxed social distancing rules for a given level of demand. Across all demand levels, best performance increases on average by 38% when moving from 2 m to 1 m social distancing. This is because more passengers are able to board thus leading to increased net economic benefits to users and increased revenue for operators at the same operational cost.

As mentioned in the “Case study - Victoria line, London underground” section, the regular operational scheme on the Victoria line during the PM peak period corresponds to a base moving block scheme with a headway of 100 s. In Table 1, we compare the best performing schemes with the regular operational scheme on the Victoria line for each demand and social distancing level. Furthermore, we include a comparison of the best performing schemes with the regular operational configuration on the Victoria line during non-pandemic conditions; the operational scheme is the base moving block scheme with a 100 s headway, the train capacity is modelled at the full capacity of 1000 passengers for Victoria line rollingstock, and demand levels are set to 100% of 2019 demand levels.

As shown in the table, across all best performing schemes, the best performing headway is not equivalent to the regular operating headway of the Victoria line of 100 s. Moreover, the best performing schemes determined as per our analysis yield better performance in all demand and train capacity scenarios when compared to the base moving block scheme under pandemic conditions for the different demand and train capacity scenarios. The improvement in the multidimensional performance criterion ranges from 12.3–195.7%, with an average improvement of 78.6% across all demand and train capacity scenarios.

When compared to the non-pandemic situation where demand is 100% of 2019 values and train capacity is a maximum of 1000 passengers, the best performance level for a 100% demand level and 2m social distancing (180 passenger capacity) is approximately 380% lower. This result is as expected, the 2 m social distancing results in a 82% reduction in capacity compared to non-pandemic operations. However, when we compare the non-pandemic performance with a 1 m social distancing scenario (290 passenger capacity), the 1 m social distancing scenario actually yields a 58% higher performance level. This result may be a consequence of the spare train capacity not being utilised efficiently in non-pandemic conditions; the tradeoff between the number of trains run and associated cost of this may outweigh the number of passengers that arrive and build up at station platforms within a 100 s headway.

Indeed, undertaking further analysis, we find that the best performing headway for the non-pandemic scenario (1000 passenger capacity and 100% of 2019 demand) is 160 s. We note that this headway is the longest headway value out of all best-performing schemes during the pandemic, and longer than the regular 100 s headway used during non-pandemic conditions. This result is as expected; in non-pandemic conditions when train capacities are being utilised at their maximum, much fewer trains are required to be run to meet demand levels. Comparing the pandemic scenarios to the 160 s headway for the non-pandemic scenario, the social distancing scenarios both perform markedly worse as expected, with a −478% and −110% reduction in performance compared to the non-pandemic scenario for 2 m and 1 m social distancing train capacities, respectively.

**Table 1:** Best performing scenario vs regular operations, baseline crowding multiplier of 1.

Train capacity	Demand (%)$$^1$$	Scenario	Headway (s)	Max. MDP ($)	Pandemic scheme MDP ($)$$^2$$	% change	Non-pandemic scheme MDP ($)$$^3$$	% change
180	35	MB	110	8.59E+04	7.47E+04	14.94		
	50	MB	70	1.29E+05	7.24E+04	78.44		
	75	MB+25D	60	2.20E+05	7.42E+04	195.71		
	100	MB+35D	60	2.01E+05	7.86E+04	155.44	3.24E+05	−379.53
290	35	FB	150	1.06E+05	8.31E+04	27.27		
	50	MB+25D	120	1.58E+05	1.41E+05	12.30		
	75	MB	80	2.45E+05	1.53E+05	60.06		
	100	MB+25D	70	3.43E+05	1.85E+05	84.77	3.24E+05	58.41

* MB* base moving block,* MB+25D* moving block with 25 s minimum dwell time,* MB+35D* moving block with 35 s minimum dwell time, * FB* fixed block.

^1^Demand is reported as a percentage of 2019 demand levels, ^2^The scheme used during the pandemic is the base moving block and 100 s headway, ^3^The scheme used during non-pandemic times is the base moving block and 100 s headway with a 1000 passenger train capacity.

### Impact of crowding multipliers

Table 2 provides a summary of the best performing operational scheme, with the associated headway and multidimensional performance values for each demand and social distancing level, for the 4 values of the additional Covid-19 multiplier applied to the platform and train crowding multipliers: (i) a baseline value of 1, (ii) multiplier of 0, (ii) multiplier of 2, and (iv) multiplier of 5.

Comparing across the four levels of the Covid-19 crowding multiplier, the results show that increasing the multiplier from a minimum of 0 to 5 has a relatively low impact on the resulting performance of the best operational schemes. On average, across all social distancing and demand levels, there is a reduction of approximately 8.4% in best performance when the crowding multiplier is inflated from 0 to 5. Plots of all crowding multiplier levels per demand and train capacity scenario for each operational scheme are included in Appendix B. As shown, the patterns of performance tend to be similar across all values of the crowding multipliers. The aforementioned results indicate that optimal supply-side decisions can neutralise a significant fraction of the user cost of crowding sensitivity due to Covid-19. As mentioned previously, it is difficult to ascertain passenger perceptions of crowding during the Covid-19 pandemic, and further work in estimating a more accurate crowding multiplier value is recommended for future work.

**Table 2:** Best performing operational scenarios by train capacity, demand level, and Covid-19 crowding multiplier.

Covid-19 multiplier	Train capacity	Demand (%)$$^1$$	Scenario	Headway (s)	MDP ($)
1	180	35	MB	110	8.59E+04
		50	MB	70	1.29E+05
		75	MB+25D	60	2.20E+05
		100	MB+35D	60	2.01E+05
	290	35	FB	150	1.06E+05
		50	MB+25D	120	1.58E+05
		75	MB	80	2.45E+05
		100	MB+25D	70	3.43E+05
0	180	35	MB	110	8.92E+04
		50	MB	80	1.21E+05
		75	MB+25D	60	2.02E+05
		100	MB+35D	70	2.09E+05
	290	35	FB	160	1.07E+05
		50	MB	110	1.62E+05
		75	MB+25D	90	2.47E+05
		100	MB+25D	60	3.30E+05
2	180	35	MB	100	8.26E+04
		50	MB	60	1.28E+05
		75	MB+25D	60	1.88E+05
		100	MB+35D	60	2.08E+05
	290	35	FB	160	1.06E+05
		50	MB	110	1.68E+05
		75	MB+25D	80	2.37E+05
		100	MB+25D	60	3.27E+05
5	180	35	MB	100	8.25E+04
		50	MB	80	1.34E+05
		75	MB+25D	60	1.84E+05
		100	MB+25D	60	2.24E+05
	290	35	FB	160	1.06E+05
		50	MB	110	1.62E+05
		75	MB+25D	80	2.35E+05
		100	MB+25D	60	3.27E+05

*MB* base moving block, *MB+25D* moving block with 25 s minimum dwell time, *MB+35D* moving block with 35 s minimum dwell time, *FB* fixed block.

^1^Demand is reported as a percentage of 2019 demand levels.

### Returns to density effects for demand levels

To investigate potential returns to density effects in terms of demand, we have calculated the value of the multidimensional performance indicator ($) normalised by demand levels (% of 2019 demand levels). A series of plots for each operational scenario for all demand and social distancing levels for a baseline crowding multiplier of 1 are included in Appendix C. A summary of the results, which shows the best performing headway and demand level per operating scheme is given in Table 3.

As shown in the figures and table, the results are mixed. The best performing headway value corresponds to each of the four different demand levels in 2 cases each. If there was an increasing returns to density effect, we would expect to see that the best performing headway value across all operational scenarios would correspond to the highest demand level of 100% of 2019 demand levels only. However, the results for the best performing scenario are mixed and do not support any distinct returns to density effect (either increasing, decreasing, or constant returns to density) in terms of demand.

**Table 3:** Performance normalised by demand - best performing headway value for each operating scheme for baseline crowding multiplier of 1.

Train capacity	Scenario	MDP/demand ($\$ / \%)$	Headway (s)	Demand level (%)$^1$
180	MB	2582	70	50
	MB+25D	2927	60	75
	MB+35D	2538	70	75
	FB	1563	140	35
290	MB	3404	60	100
	MB+25D	3426	70	100
	MB+35D	3136	110	50
	FB	3022	150	35

*MB* base moving block,* MB+25D* moving block with 25 s minimum dwell time,* MB+35D* moving block with 35 s minimum dwell time,* FB* fixed block

^1^Demand is reported as a percentage of 2019 demand levels.

### Comparison across different operating schemes

In the following sections, we analyse the performance of each operational scenario for a baseline crowding multiplier of 1. We compare the performance of each scenario with the regular performance configuration of the Victoria line during pandemic conditions, which is a base moving block scheme operated at a 100 s headway.

#### Moving block

The base moving block scheme performs best in 3 out of 8 demand and social distancing cases. In the base moving block scheme, separation distances between trains are minimised and dwell times on platforms are shorter; therefore more trains can be run to serve demand while minimising train crowding. On average across all demand and social distancing scenarios, performance gains of approximately 60.2% are achieved when comparing the best performing headway for the moving block scheme with the regular operating scheme of the Victoria line during pandemic conditions.

#### Moving block with longer minimum dwell times

The moving block scheme with a 25 s minimum dwell time performs best across 3 out of 8 cases tested, while the moving block scheme with a 35 s minimum dwell time performs best in 1 case. Generally, the moving block schemes with longer minimum dwell times of 25 s and 35 s tend to perform better than the base moving block scheme at higher demand levels. This is likely a result of more passengers being able to board when the dwell time is longer, and that this effect becomes more advantageous at higher demand levels. At lower demand levels, it is likely that having longer dwell times is not beneficial, as all passengers waiting on the platform are able to board within a shorter dwell time, and the additional dwell time is lost time.

When comparing the best performing headway of the longer dwell time schemes with the regular scheme on the Victoria line, the moving block scheme with a minimum 25 s dwell time performs 70.3% better than the regular scheme and the moving block scheme with a minimum 35 s dwell time performs 66.2% better than the regular scheme on average across all demand and social distancing scenarios.

#### Fixed block

The fixed block signalling scheme is the best performing scheme for one scenario, when demand is at 35% of 2019 levels and the train capacity is 290 passengers. However, across all other scenarios, the fixed block scheme performs worst. The fixed block scheme requires the greatest separation distances between trains, which can lead to train delays and train bunching particularly at shorter headways, thus leading to longer passenger wait times, and higher levels of crowding on platforms. At the lowest demand level with the highest train capacity, the savings from running fewer trains with the fixed block scheme outweighs the penalties from longer wait times and crowding, thus the fixed block scheme performs best in this scenario. However, in all other cases, the penalties for longer wait times and crowding are more prominent, hence the fixed block scheme’s poor performance in all other cases. Averaged across all demand levels and distancing levels, the best performing headway for the fixed block scheme is 11% lower than the regular scheme on the Victoria line.

### Comparison with other performance criteria

So far, we have presented the best performing headway calculated as per the multidimensional performance criterion defined in Eq. (7). As our simulation modelling platform is flexible, we can also evaluate the different operational schemes with respect to other performance criteria. Here, in addition to the multidimensional performance criterion, we present the best performing schemes for each demand and social distancing scenario in terms of the following criteria:

- Average total number of Covid-19 cases - We estimate the number of Covid-19 cases for each scenario using the well known Wells-Riley equation for airborne disease transmission in enclosed spaces. We take the standard approach for undertaking comparative analyses and assume that there is one infectious person at the platform and in the train at any given time. We then calculate the average infection probability at each platform between train arrivals, and the average infection probability aboard each train. The number of cases is calculated as the infection probability multiplied by the number of passengers in the space of concern. The average total number of Covid-19 cases for each operational scenario is the sum of the potential average number of cases per platform and per train. The calculations are summarised in Appendix D. The operational scheme and headway value which has the minimum number of Covid-19 cases is taken as the best performing. It should be noted that there is a high degree of uncertainty in the virus transmission factors, with potential uncertainty up to a factor of 5^37^. Furthermore, we do not consider higher order effects of potential transmission when passengers interact during boarding and alighting, as demonstrated for example by Qian et al.^38^ in their quantification of infection risk through the construction of individual level contact networks. Therefore, the number of Covid-19 cases calculated here should not be taken as an accurate absolute estimate, but should be interpreted as a relative measure to compare different operational schemes.
- Profit ($) - Profit is calculated as the difference between revenue from ticket prices from boarding passengers and the costs of running trains as per Eq. (9). The operational scheme and headway value which has the greatest profit is taken as best performing.
- Total journey time (s) - Total journey time is defined as the the sum of journey times across all passengers who make the decision to board. Journey times at an individual passenger level are the sum of a passenger’s in-vehicle time as per Eq. (4) and wait time as per Eq. (3). The operational scheme and headway value which has the shortest total journey time is taken as the best performing.
- Maximum number of passengers per platform - For each headway scenario, the maximum number of passengers that build up on each platform is calculated, and then the maximum value across all platforms is taken as the maximum number of passengers per platform for that scenario. The operational scheme and headway that performs best is that which has the minimum number of maximum passengers per platform.

**Figure 3:**
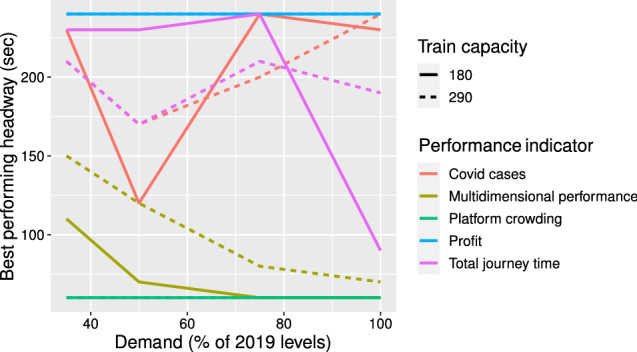
Best performing headway for different performance criteria for baseline Covid-19 multiplier of 1.

Table 4 and Figure 3 illustrate the best performing headway results for each performance criteria by train capacity and demand level for a baseline Covid-19 multiplier of 1. Please note that the results for the MDP criterion have been previously presented in Tables 1 and 2, and so are not included in Table 4 to avoid repetition.

**Table 4:** Summary of best performing headway and operational scheme for different performance criteria per train capacity and demand level, for baseline Covid-19 crowding multiplier of 1.

Train capacity	Demand (%)$$^1$$	Scenario	Headway (s)	Min. value
*Total Covid-19 cases*				
180	35	MB+25D	230	2.08E−01
	50	MB+35D	120	2.41E−01
	75	MB+35D	240	2.68E−01
	100	MB+35D	230	3.07E−01
290	35	MB	210	2.76E−01
	50	MB+35D	170	3.08E−01
	75	MB+25D	200	3.49E−01
	100	MB+35D	240	4.42E−01
*Profit ($)*				
Train capacity	Demand (%)$$^1$$	Scenario	Headway (s)	Max. value
180	35	MB+35D	240	−2.81E+04
	50	MB+35D	240	−2.82E+04
	75	MB+35D	240	−2.79E+04
	100	MB+35D	240	−2.71E+04
290	35	MB+35D	240	−2.80E+04
	50	MB+35D	240	−2.70E+04
	75	MB+35D	240	−2.65E+04
	100	MB+35D	240	−2.59E+04
*Total journey time (s)*				
Train capacity	Demand (%)$$^1$$	Scenario	Headway (s)	Min. value
180	35	MB+25D	230	3.48E+05
	50	MB+25D	230	1.36E+05
	75	MB+35D	240	1.92E+04
	100	FB	90	9.70E+03
290	35	MB	210	7.04E+05
	50	MB	170	7.64E+05
	75	MB+35D	210	1.31E+04
	100	MB+35D	190	7.56E+03
*Maximum number of passengers on platform*				
Train capacity	Demand (%)$$^1$$	Scenario	Headway (s)	Min. value
180	35	MB+25D	60	28
	50	MB+25D	60	39
	75	MB+35D	60	86
	100	MB+25D	60	307
290	35	MB+25D	60	26
	50	MB	60	41
	75	MB+25D	60	63
	100	MB	60	85

*MB* base moving block,* MB+25D* moving block with 25 s minimum dwell time,* MB+35D* moving block with 35 s minimum dwell time,* FB* fixed block

^1^Demand is reported as a percentage of 2019 demand levels.

As seen in the figure and table, there is not a single consistent value of headway or operational scheme which yields the best performance across all criteria and all demand and social distancing scenarios. As previously discussed, the best performing headway according to the MDP decreases as demand increases and as train capacity decreases. The best performing headway for profit and platform crowding is constant over all demand and train capacity scenarios. For profit, the best performing scheme is that which has the longest headway. This reflects the high cost of running trains; the highest profit is attained when the fewest trains are run in all scenarios tested here. For platform crowding, the best performing headway is the shortest. This is as expected; the fewest passengers build up at station platforms when the headway between trains is shortest. For the total journey time and Covid-19 cases indicators, there is no clear pattern. For total journey time, the best performing headway ranges from 90–230 s for a 180 passenger train capacity and 170–210 s for a 290 passenger train capacity. For the number of Covid-19 cases, the best performing headway ranges from 120–240 s for a train capacity of 180 passengers and 170–240 s for a train capacity of 290 passengers.

As a result of the wide dispersion across the different performance criteria, the results clearly highlight that the selection of the performance criteria is an important factor in determining the best performing operational scheme. It is typically at the operator’s discretion to determine their target priorities and hence specify the performance criteria which best reflects this. In this paper, we have focused on evaluating performance as per the MDP indicator as we believe that it is well suited to the general evaluation of public goods such as public transport. During pandemic conditions, it would indeed be pertinent to include the risk of virus transmission in the calculation of MDP, however, as mentioned previously, the Covid-19 disease dynamics have been subject to rapid change throughout the pandemic and it has been difficult to estimate correct values of the transmission factors in the calculations; moreover, it is difficult to assign a monetary cost of Covid-19 cases for direct inclusion in the MDP indicator. In the future, if more reliable information becomes available for the transmission dynamics of Covid-19 and valuing the cost of Covid-19 cases, then we recommend to expand the MDP indicator to include the effect of Covid-19 cases directly.

## Conclusion

In this paper, we present a flexible and tractable agent-based simulation platform to evaluate the performance of mass public transport networks during the Covid-19 pandemic. Using the Victoria Line on the London Underground as a case study, we demonstrate a range of potential operational solutions for varied demand levels under 2 m and 1 m social distancing restrictions. The advantage of the flexible and tractable modelling platform that we have developed is that a wide range of operational solutions can be trialled under a number of different demand and social distancing requirements. This capability is not necessarily currently available to transit operators, who typically possess complex and intractable in-house modelling platforms calibrated to a narrow range of operating conditions.

We trial the following operational interventions: alterations to headways, dwell times, signalling schemes, and alterations to train paths. We demonstrate that the modelling platform can be used to determine the best performing headway and operational scheme over a specified range of headways. We compare the schemes against the regular operations of the Victoria line during weekday PM periods during the pandemic, i.e. a base moving block scheme with a 15 s minimum dwell time and 100 s headway. We find that the best performing schemes across all social distancing and demand scenarios do not identify the regular 100 s headway as the best performing in any case. The best performing headways differ depending on the demand and social distancing levels, ranging from 60 to 150 s. Moreover, the results do not identify a single operational scheme that performs best consistently in all scenarios, the best scheme is again highly context dependent on demand and social distancing levels. Over all demand and social distancing scenarios, the performance gains for the best performing headway and operational scheme range from 12.3–195.7%, with an average improvement of 78.6%, when compared to the regular operations on the Victoria line during the pandemic. The results therefore highlight the need and benefit of the flexible simulation platform; multiple demand and social distancing scenarios can be simulated to achieve substantial performance gains.

In terms of comparisons across the different operational schemes, we find that the moving block schemes with varying dwell times perform similarly well and far better than the fixed block scheme, while the skip-stop scheme performs worst across all demand and social distancing scenarios. Compared to the regular operations on the Victoria line during the pandemic, when averaged across all demand and social distancing scenarios, the best performing headway for the moving block scheme with a minimum 25 s dwell time has a 70.3% higher MDP value, for the moving block scheme with a minimum 35 s dwell time the performance is 66.2% higher, and for the moving block scheme with a minimum 15 s dwell time the performance is 60.2% higher. Across all demand and social distancing levels, the best performance of the older fixed block signalling scheme is 11% lower than the regular moving block scheme used during the pandemic. Comparing the best performance of the fixed block scheme with the best performance of the moving block scheme with a minimum 25 s dwell time, the performance of the fixed block scheme is 42.3% lower. Transit authorities can therefore use the modelling platform (with inputs of the actual rather than simulated fixed block locations) to make an evidenced-based case for upgrading signalling technologies.

In terms of future work, we have identified a number of areas to explore further. First is the potential inclusion of passenger perceptions of Covid-19 risk in the generalised costs calculations and second is the direct inclusion of the social cost of Covid-19 infections in the calculation of the multidimensional performance criterion. In our analysis, we undertook sensitivity testing of passenger perceptions of crowding during pandemic conditions by trialling the application of additional multipliers to the platform and train crowding costs. We found that our results were relatively robust to crowding sensitivity, with the average change in the best performance levels being 8.4% when inflating the crowding multipliers from 0 to 5, however, we acknowledge that future work to generate more accurate estimates of crowding sensitivity would be beneficial. We also used a simplified Wells-Riley method to calculate the potential infection risk and number of Covid-19 cases that could arise in each operational scheme. However, we note that there is substantial uncertainty in the estimates of the transmission dynamics of the virus potentially up to a factor of 5 as reported in the literature^37^, we do not consider higher order individual contact effects, such as those shown earlier^38^, which could be included to improve the accuracy of infection risk estimation, and there is uncertainty in determining the societal costs of infection. Therefore, we recommend these areas for further work to potentially improve the evaluation of operational scenarios. Finally, the current model assumes unconstrained queuing when passengers are unable to board the train. In future work, the model can be expanded to incorporate demand side interventions to control queue length. Potential solutions have been documented in our paper^3^, and include ticket pricing, advanced booking, slot auctioning, and tradeable travel permit schemes.

## Supplementary Information

Supplementary Information 1.

## Data Availability

The data used in the model were obtained from the Transport for London open-source data repository, which is freely available at http://crowding.data.tfl.gov.uk.

## Code availability

The R code for the model can be made available on request.
