# Supplementary material for: An evaluation framework for operational interventions on urban mass public transport during a pandemic
Source: Sci Rep. 2023 Mar 30;13:5163. doi: 10.1038/s41598-023-31892-2 (PMC10060931; doi:10.1038/s41598-023-31892-2)
Supplement: Supplementary file 1 — Supplementary Information 1. [file 41598_2023_31892_MOESM1_ESM.pdf]

An evaluation framework for operational interventions on urban mass public transport during a pandemic

Ramandeep Singh[1], Daniel Hörcher[1] and Daniel J. Graham[1*]

[1*]Transport Strategy Centre, Centre for Transport Studies, Department of Civil and Environmental Engineering, Imperial College London, Exhibition Road, London, SW73AE, UK.

*Corresponding author(s). E-mail(s): d.j.graham@imperial.ac.uk; Contributing authors: ramandeep.singh13@imperial.ac.uk; d.horcher@imperial.ac.uk;

Appendix A Train and passenger movement modelling

A.1 Inputs

The inputs to the train movement simulation are as follows:

- Line layout - line length and position of stations
- Rolling stock properties - train length, passenger capacity, maximum speed, and acceleration and braking rates
- Passenger movements - average arrival and alighting rates at each station. Passengers are assumed to follow a random Poisson arrival process as is conventional for high frequency transit networks (refer to [1] and references therein).

A.2 Train movement rules

The following section explicitly details the train movement rules. The variables used are defined as follows:

n refers to the train number $n = 1...N$
s refers to the station number $s = 1...S$
t refers to time in seconds, i.e. $t = 1...T$ where $T = 3600$ sec
v_{nt} is the velocity of train n at time t in m/s
x_{nt} is the position of train n at time t in m, i.e. $x = 1...X$
T_n is the total time that train n has been active in the network in seconds
h is headway in seconds
$v_{\max} = 20$ m/s is the maximum train velocity
$a = 1$ m/s^2 is the acceleration of the train
$b = 1$ m/s^2 is the deceleration of the train
$d_s = 80$ m is the safety distance of the train
d_b is the braking distance of the train in m
δ is the required separation distance between trains in m as per equation **??**
τ is the distance between train n and the nearest station in m
Ω is the distance between train n and previous train $n-1$, i.e. $\Omega = x_{(n-1)t} - l - x_{nt}$
l is the train length in m
C is the maximum dwell time for the station in seconds, calculated as per equation A4
c_{st} is the dwell count at station s at time t, i.e. $c_{st} = 0, 1...C$
x_S is the position of the last station

Calculation of maximum dwell times:

$$C_\beta = \frac{P_{st}}{q_{\max} - q_\alpha} \tag{A1}$$

$$C_{R\beta} = [C_\beta, C_\beta + 5] \tag{A2}$$

$$C_{R\alpha} = [C_\alpha, C_\alpha + 5] \tag{A3}$$

$$C = max(C_{R\beta}, C_{R\alpha}) \tag{A4}$$

where C_β is the minimum dwell time to enable boardings of all passengers P_{st} at the platform for station s at time t, $q_{\max}$ is the maximum flow rate of passengers (boarding and alighting combined), q_α is the flow rate of alighting passengers. A minimum dwell time C_α is specified to enable alighting movements. $C_{R\beta}$ and $C_{R\alpha}$ are randomly sampled dwell times to accommodate boarding and alighting movements. The final maximum dwell time C at each station is allocated as the maximum value of the randomly selected boarding dwell time and randomly selected alighting time.
Train n entry into network at time t:

1. If $T_{(n-1)} \geq h$ and $x_{(n-1)t} \geq \frac{v_{\max}^2}{2b} + d_s + l$, then $v_{nt} = v_{\max}$ and $x_{nt} = 1$.

When train n is behind train $n-1$:
Velocity

1. If $\Omega > \delta$, then $v_{n,t+1} = min(v_{nt} + a, v_{\max})$.

2. Else if $\Omega < \delta$, then $v_{n,t+1} = max(v_{nt} - b, 0)$.
3. Else if $\Omega = \delta$, then $v_{n,t+1} = v_{nt}$

Position

1. $x_{n,t+1} = x_{nt} + v_{n,t+1}$

Train n is behind station occupied by train $n-1$:
Velocity

1. If $\Omega > \delta$, then $v_{n,t+1} = min(v_{nt} + a, v_{\max})$.
2. Else if $\Omega < \delta$, then $v_{n,t+1} = max(v_{nt} - b, 0)$.
3. Else if $\Omega = \delta$, then $v_{n,t+1} = v_{nt}$

Position

1. $x_{n,t+1} = x_{nt} + v_{n,t+1}$

Train n is behind empty station:
Velocity

1. If $\tau > d_b$, then $v_{n,t+1} = min(v_{nt} + a, v_{\max})$.
2. Else if $\tau < d_b$, then $v_{n,t+1} = max(v_{nt} - b, 0)$, and then $v_{n,t+1} = min(v_{n,t+1}, \sqrt{2b\tau})$ (deceleration)
3. Else if $\tau = d_b$, then $v_{n,t+1} = v_{nt}$

Position

1. $x_{n,t+1} = x_{nt} + v_{n,t+1}$

Train n is at station:
Velocity and dwell count

1. If $c_t = C$ and $\Omega > \delta$, then $v_{n,t+1} = min(v_{nt} + a, v_{\max})$ and $c_{t+1} = 0$
2. Else if $c_t = C$ and $\Omega < \delta$, then $v_{n,t+1} = 0$ and $c_{t+1} = 0$
3. Else if $c_t < C$, then $v_{n,t+1} = 0$ and $c_{t+1} = c_t + 1$

Position

1. $x_{n,t+1} = x_{nt} + v_{n,t+1}$

Train n is beyond the last station in the network:
Velocity

1. If $x_{nt} > x_S$ and $x_{n-1,t} \geq X$, then $v_{n,t+1} = min(v_{nt} + a, v_{\max})$.
2. Else if $x_{nt} > x_S$ and $x_{n-1,t} < X$,
   and if $\Omega > \delta$, then $v_{n,t+1} = min(v_{nt} + a, v_{\max})$
   else if $\Omega < \delta$, then $v_{n,t+1} = max(v_{nt} - b, 0)$.
   else if $\Omega = \delta$, then $v_{n,t+1} = v_{nt}$.

Position

1. $x_{n,t+1} = min(x_{nt} + v_{n,t+1}, X)$

Velocity

1. Else if $x_{nt} \geq X$, then $v_{n,t+1} = min(v_{nt} + a, v_{\max})$.

Position

1. $x_{n,t+1} = X$

A.3 Passenger movement rules

The variables and rules for passenger movement are defined in the following section.

q_γ is the passenger arrival rate in passengers/sec at time t
q_α is the passenger alighting rate in passengers/sec at time t
q_β is the passenger boarding rate in passengers/sec at time t
q_χ is the maximum passenger boarding rate in passengers/sec at time t
$q_{\max}$ is the maximum flow rate of passenger movements in passengers/sec, i.e. $q_{\max} = q_\beta + q_\alpha$
P_{st} is the number of passengers at the platform of station s at time t
M is the maximum train capacity (number of passengers)
m_{nt} is the number of passengers on train n at time t, i.e. $m_{nt} = 0, 1..M$
$z_{n,t+1}$ is the number of passengers admitted on the train when the number of passengers on the train is at maximum at $t+1$

If $P_{st} + q_\alpha < q_{\max}$

1. Passengers on train
   $m_{n,t+1} = min(m_{nt} + P_{st} - q_\alpha, M)$ and $m_{n,t+1} = max(m_{n,t+1}, 0)$.
2. Passengers on platform
   If $m_{n,t+1} < M$, then $q_\beta = P_{st}$ and $P_{s,t+1} = q_\gamma$
   Else if $m_{n,t+1} = M$, then $z_{n,t+1} = M - (m_{nt} - q_\alpha)$ and $q_\beta = z_{n,t+1}$ and $P_{s,t+1} = P_{st} - q_\beta + q_\gamma$

If $P_{st} + q_\alpha \geq q_{\max}$

1. Passengers on train
   $q_\chi = q_{\max} - q_\alpha$ and $m_{n,t+1} = min(m_{nt} + q_\chi - q_\alpha, M)$ and $m_{n,t+1} = max(m_{n,t+1}, 0)$.

2. Passengers on platform
   If $m_{n,t+1} < M$, then $q_\beta = q_\chi$ and $P_{s,t+1} = P_{st} - q_\beta + q_\gamma$
   Else if $m_{n,t+1} = M$, then $z_{n,t+1} = M - (m_{nt} - q_\alpha)$ and $q_\beta = z_{n,t+1}$ and $P_{s,t+1} = P_{st} - q_\beta + q_\gamma$

A.4 Outputs

Two classes of outputs are generated from the train movement simulation, relating to trains and stations, respectively. At each time step t, the following quantities are output:

i. Train n - Position x_{nt}, velocity v_{nt}, number of passengers on board m_{nt}, and train time counter c_{nt}
ii. Station s - Number of passengers on platform P_{st}, arrivals $q_{\gamma st}$, boards $q_{\beta st}$, alights $q_{\alpha st}$, dwell time counter c_{st}

Appendix B Results on all crowding multipliers

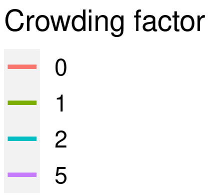

Fig. B1: Legend for Figures B2 to B9

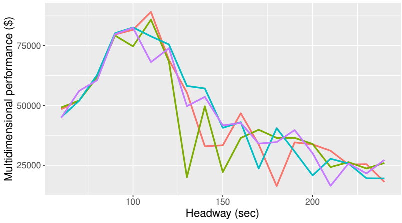

(a) 35% demand level

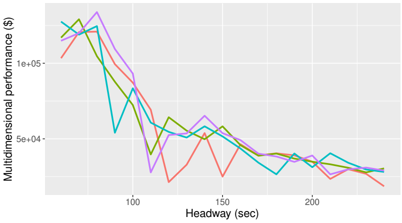

(b) 50% demand level

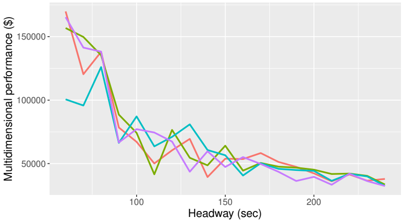

(c) 75% demand level

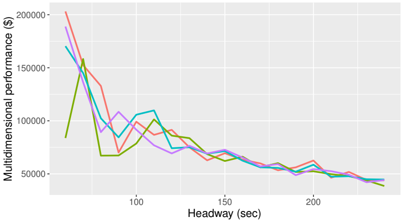

(d) 100% demand level

Fig. B2: Performance vs headway for base moving block, all crowding multipliers, train capacity of 180 passengers

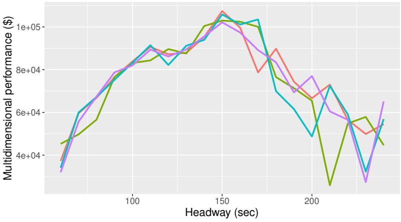

(a) 35% demand level

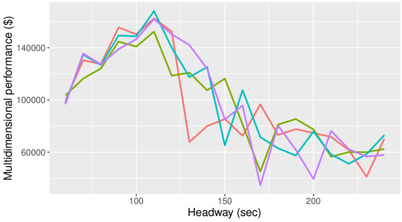

(b) 50% demand level

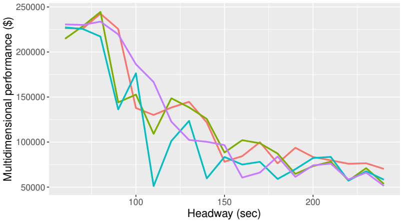

(c) 75% demand level

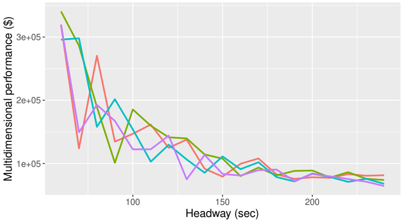

(d) 100% demand level

Fig. B3: Performance vs headway for base moving block, all crowding multipliers, train capacity of 290 passengers

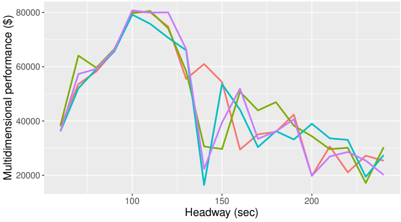

(a) 35% demand level

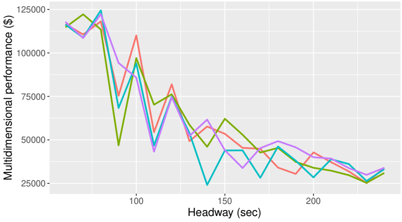

(b) 50% demand level

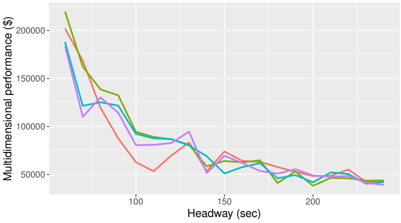

(c) 75% demand level

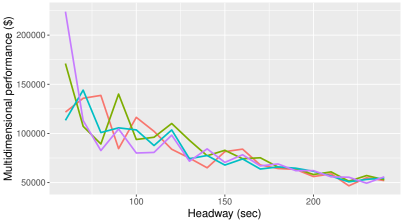

(d) 100% demand level

Fig. B4: Performance vs headway for moving block with 25 sec minimum dwell time, all crowding multipliers, train capacity of 180 passengers

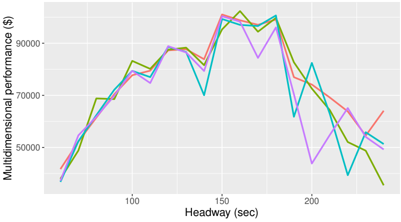

(a) 35% demand level

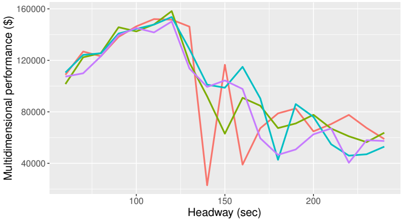

(b) 50% demand level

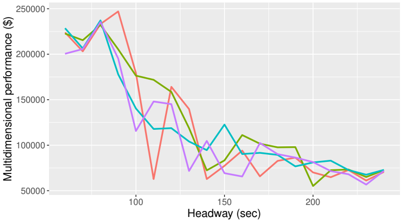

(c) 75% demand level

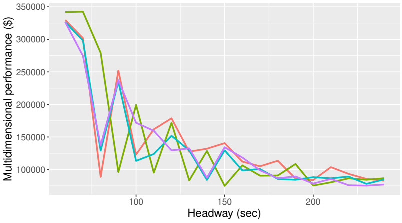

(d) 100% demand level

Fig. B5: Performance vs headway for moving block with 25 sec minimum dwell time, all crowding multipliers, train capacity of 290 passengers

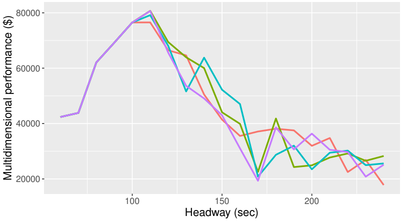

(a) 35% demand level

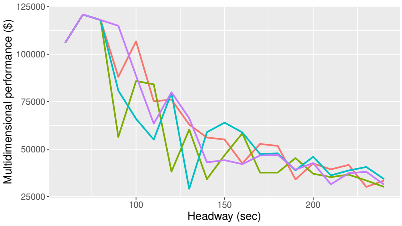

(b) 50% demand level

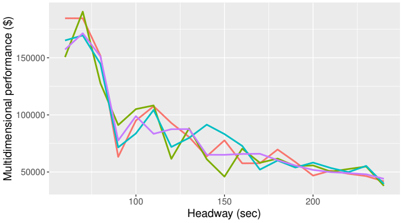

(c) 75% demand level

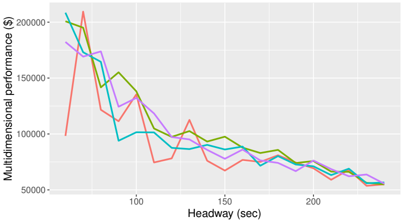

(d) 100% demand level

Fig. B6: Performance vs headway for moving block with 35 sec minimum dwell time, all crowding multipliers, train capacity of 180 passengers

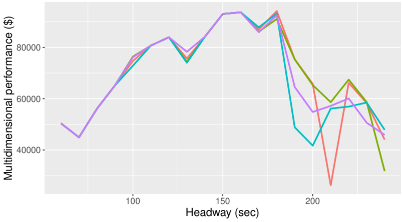

(a) 35% demand level

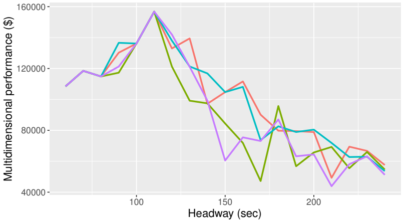

(b) 50% demand level

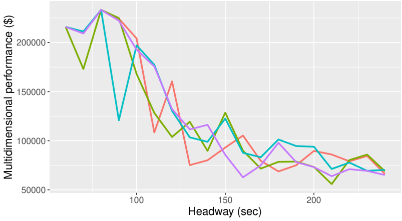

(c) 75% demand level

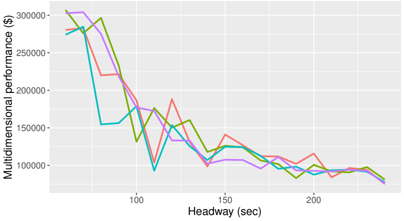

(d) 100% demand level

Fig. B7: Performance vs headway for moving block with 35 sec minimum dwell time, all crowding multipliers, train capacity of 290 passengers

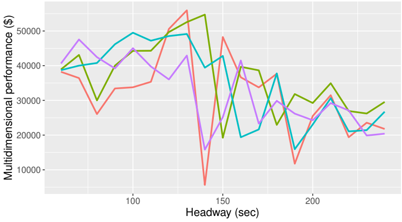

(a) 35% demand level

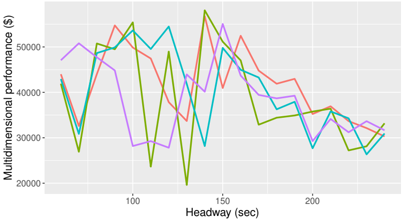

(b) 50% demand level

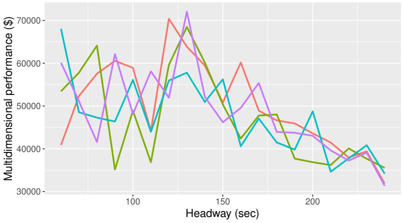

(c) 75% demand level

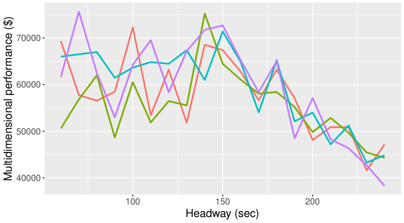

(d) 100% demand level

Fig. B8: Performance vs headway for fixed block, all crowding multipliers, train capacity of 180 passengers

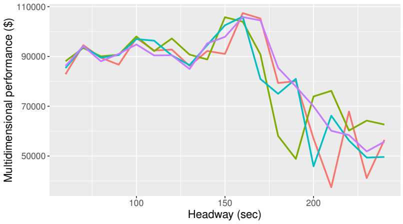

(a) 35% demand level

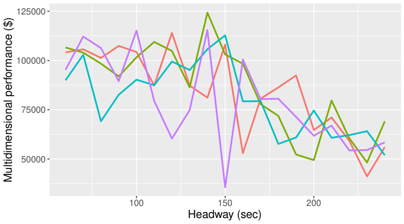

(b) 50% demand level

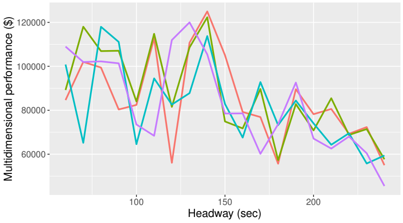

(c) 75% demand level

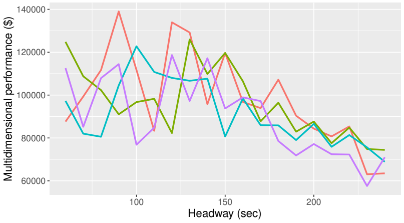

(d) 100% demand level

Fig. B9: Performance vs headway for fixed block, all crowding multipliers, train capacity of 290 passengers

Appendix C Results on normalised multidimensional performance

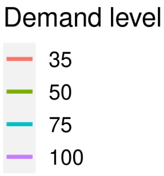

Fig. C10: Legend for Figures C11 and C12

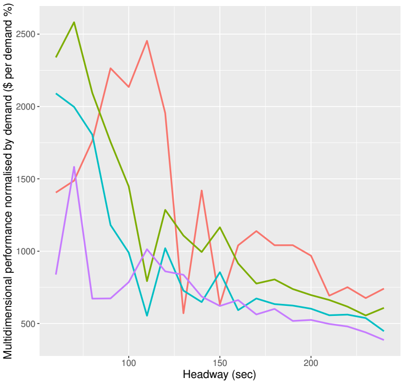

(a) Moving block base

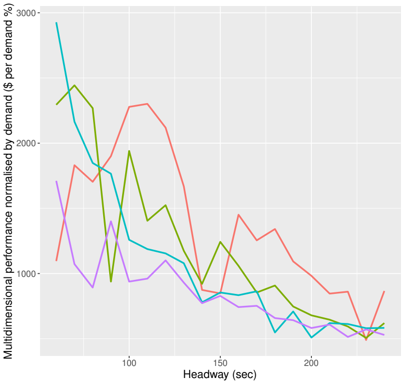

(b) Moving block + 25 sec min dwell

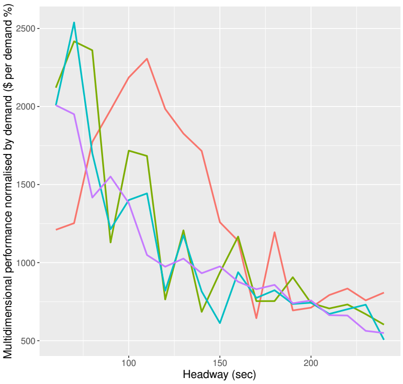

(c) Moving block + 35 sec min dwell

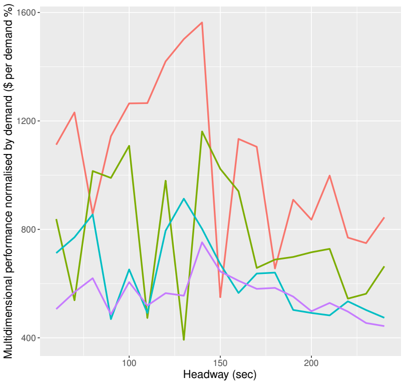

(d) Fixed block

Fig. C11: Performance normalised by demand vs headway, baseline crowding multiplier of 1, train capacity of 180 passengers

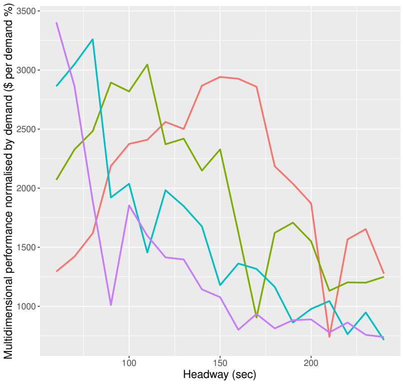

(a) Moving block base

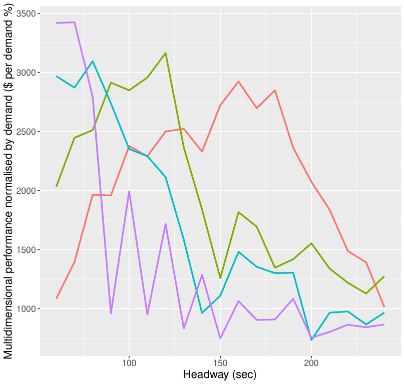

(b) Moving block + 25 sec min dwell

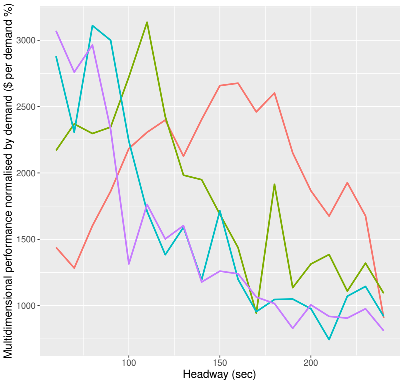

(c) Moving block + 35 sec min dwell

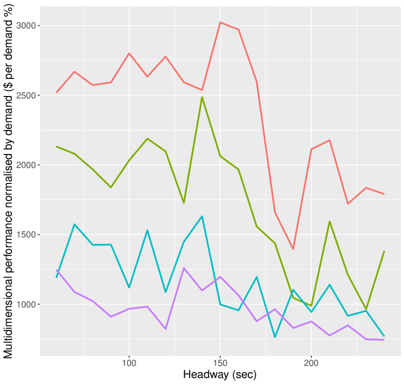

(d) Fixed block

Fig. C12: Performance normalised by demand vs headway, baseline crowding multiplier of 1, train capacity of 290 passengers

Appendix D Calculations of Covid-19 infection risk

It is becoming widely accepted that SARS-CoV-2, the virus pathogen that causes the Covid-19 disease, is transmitted through airborne transmission [2]. The most commonly used model to estimate airborne transmission of viruses in enclosed spaces is the Wells-Riley model [3, 4]. Here we use the Wells-Riley model to estimate the risk of infection in the enclosed environment of the Victoria line on the London Underground using the calculation methodology presented in [2], [5], [6], and [7] as a guide.

It is very difficult to obtain concise estimates of cases as the dynamics of the SARS-CoV-2 pathogen and the Covid-19 disease are subject to a high degree of uncertainty. For example, [2] states in their estimation tool that the estimate used to measure the quanta (infective dose) exhalation rate (q_{ex}) could

have uncertainty up to the order of a factor of 5. The multiplication factor representing the tranmissibility of different variants of the virus is also associated with a high degree of uncertainty. For this reason, we use a simplified process to obtain average estimates of cases, as the primary aim of the analysis is to compare operational schemes, and thus we are more concerned with relative performance rather than obtaining concise absolute estimates of potential cases.

We generate separate estimates of potential Covid-19 cases when passengers are waiting in the underground station platform environment and when passengers are travelling on the train. For station platforms, we calculate the average number of cases generated at every platform in time periods that represent every consecutive time gap between successive trains departing the platform. For trains, we calculate the average number of cases throughout the whole train run from the first station to the last station. As we are assessing relative performance, we make the standard assumption that there is one infectious passenger. The total number of cases for each operational scenario is the sum of the cases calculated across all platforms and all trains. It should be noted that here we consider first order airborne transmission impacts only, and do not consider potential higher-order impacts which may arise when passengers exposed to different infection risk levels mix when entering and leaving the train and on the train. The inclusion of higher order effects, as demonstrated for example by [8] in their quantification of individual level contact networks, is recommended as an area of future work.

We use the most recent estimates for the variables representing disease transmission and vaccination rates. Furthermore, mask restrictions were legally enforced on the London Underground from 15 June 2020 to 18 July 2021 [9]. However, as the parameters for mask inhalation and exhalation efficiency are subject to a high degree of uncertainty, we have taken the conservative view and have not included any potential inhibiting effects that facial masks may have on virus transmission. The process for estimating the number of Covid-19 cases at each station platform between consecutive trains and on a train is detailed in the following sections.

First, we calculate the quanta exhalation rate q_{ex}, which is measured in infective doses per hour i.e. quanta h^{-1}:

$$q_{\mathrm{ex}} = q_b \delta \alpha \tag{D5}$$

where q_b refers to the basic quanta exhalation rate (quanta h^{-1}), which is assumed to be 18.6 quanta h^{-1} for standing and breathing activity as per [2]. δ is a multiplication factor to account for SARS-CoV-2 virus variants, which is conservatively assumed to be 3.3 for the most transmissible variant Omicron BA.2 as per [10] and [2]. α is a multiplication factor for physical activity and vocalisation, which is taken here as 1.2 as per [2].

Then the net emission rate (quanta h^{-1}) is calculated as follows:

$$E_n = q_{\mathrm{ex}} * n_i \tag{D6}$$

where n_i is the number of infected persons, which is taken in all calculations as a baseline of 1 person as previously mentioned.

The total first order loss rate λ (h^{-1}) is calculated as:

$$\lambda = \lambda_v + \lambda_d + \lambda_s \tag{D7}$$

where λ_v is the rate of ventilation with outside air (h^{-1}). For platforms on the Victoria line, this value is 10 h^{-1} and for Victoria line rollingstock this value is 33 h^{-1} [11]. λ_d is the decay rate of the virus, taken as 0.62 h^{-1} as per [12] and [2]. λ_s is the surface deposition rate, taken as 0.3 h^{-1} as per [2], [5], [6], and [7].

The average quanta concentration c (quanta m^{-3}) is calculated as:

$$c = \frac{E_n}{\lambda/V} * \left(1 - \frac{1}{\lambda/t}\right) * \left(1 - e^{-\lambda t}\right) \tag{D8}$$

where V is the volume of the enclosed space (m^3). For the station platform tube tunnel, this is 2329.669 m^3 [13, 14], and for Victoria line train carriages, this is 123.2728 m^3 as per [15]. t is the exposure time in hours (h). For the calculations at the station platform, the exposure time is calculated as the average wait time of passengers waiting between consecutive train arrivals. For the calculation of cases in the train, the exposure time is calculated as the average in-vehicle time of passengers on the train.

The quanta inhaled per person q_p is measured in quanta and calculated as:

$$q_p = c * B * t \tag{D9}$$

where B is the breathing rate of susceptible persons (m^3/h). This is assumed to be 0.615 m^3/h which is the mid-range of short-term exposure values for light to moderate intensity activity as per [16] and [2].

We then calculate the probability of infection P as follows:

$$P = 1 - e^{-q_p} \tag{D10}$$

The number of susceptible persons n_s is calculated as:

$$n_s = (n_t - n_i) * (1 - \gamma) \tag{D11}$$

where n_t is the number of persons in the enclosed space. For the calculations at the station platforms, this is calculated as the average number of passengers waiting between consecutive train arrivals. For the calculation of cases in the train, this is calculated as the average number of passengers in one train carriage. We make the assumption that TfL enforces social distancing such that passengers are dispersed evenly into the 8 train carriages of the Victoria Line rollingstock. γ is the fraction of the population which is immune. We take this as 0.882, which corresponds to 88.2% of the population in London being fully vaccinated with their second dose as of 28 September 2022 as per [17].

Finally, the number of estimated Covid-19 cases n_c is calculated as:

$$n_c = P * n_s \tag{D12}$$

References

[1] Singh, R., Graham, D.J., Hörcher, D., Anderson, R.: The boundary between random and non-random passenger arrivals: Robust empirical evidence and economic implications. Transportation Research Part C: Emerging Technologies **130**, 103267 (2021)

[2] Peng, Z., Rojas, A.L.P., Kropff, E., Bahnfleth, W., Buonanno, G., Dancer, S.J., Kurnitski, J., Li, Y., Loomans, M.G.L.C., Marr, L.C., Morawska, L., Nazaroff, W., Noakes, C., Querol, X., Sekhar, C., Tellier, R., Greenhalgh, T., Bourouiba, L., Boerstra, A., Tang, J.W., Miller, S.L., Jimenez, J.L.: Practical indicators for risk of airborne transmission in shared indoor environments and their application to COVID-19 outbreaks. Environmental Science and Technology **56**, 1125–1137 (2022)

[3] Wells, W.F.: Airborne Contagion and Air Hygiene: An Ecological Study of Droplet Infections. Harvard University Press (for The Commonwealth Fund), Cambridge, MA (1955)

[4] Riley, E.C., Murphy, G., Riley, R.L.: Airborne spread of measles in a suburban elementary school. American Journal of Epidemiology **107**(5), 421–432 (1978)

[5] Miller, S.L., Nazaroff, W.W., Jimenez, J.L., Boerstra, A., Buonanno, G., Dancer, S.J., Kurnitski, J., Marr, L.C., Morawska, L., Noakes, C.: Transmission of SARS-CoV-2 by inhalation of respiratory aerosol in the Skagit Valley Chorale superspreading event. Indoor Air **31**(2), 314–323 (2021)

[6] Buonanno, G., Stabile, L., Morawska, L.: Estimation of airborne viral emission: Quanta emission rate of SARS-CoV-2 for infection risk assessment. Environment International **141**, 105794 (2020)

[7] Buonanno, G., Stabile, L., Morawska, L.: Quantitative assessment of the risk of airborne transmission of SARS-CoV-2 infection: Prospective and retrospective applications. Environment International **145**, 106112 (2020)

[8] Qian, X., Sun, L., Ukkusuri, S.V.: Scaling of contact networks for epidemic spreading in urban transit systems. Scientific Reports **11**, 4408 (2021)

[9] Department for Transport: The Health Protection (Coronavirus, Wearing of Face Coverings on Public Transport) (England) Regulations 2020 (revoked). Accessed at www.legislation.gov.uk/uksi/2020/592/introduction (2021)

[10] Lyngse, F.P., Kirkeby, C.T., Denwood, M., Christiansen, L.E., Mølbak, K., Møller, C.H., Skov, R.L., Krause, T.G., Rasmussen, M., Sieber, R.N., Johannesen, T.B., Lillebaek, T., Fonager, J., Fomsgaard, A., Møller, F.T., Stegger, M., Overvad, M., Spiess, K., Mortensen, L.H.: Household transmission of SARS-CoV-2 Omicron variant of concern subvariants BA.1 and BA.2 in Denmark. Nature Communications **13**(5760), 1–7 (2022)

[11] Department for Transport: FOI request detail: Air refresh. Accessed at tfl.gov.uk/corporate/transparency/freedom-of-information/foi-request-detail?referenceId=FOI-0219-2122 (2021)

[12] van Doremalen, N., Bushmaker, T., Morris, D.H., Holbrook, M.G., Gamble, A., Williamson, B.N., Tamin, A., Harcourt, J.L., Thornburg, N.J., Gerber, S.I., Lloyd-Smith, J.O., de Wit, E., Munster, V.J.: Aerosol and Surface Stability of SARS-CoV-2 as Compared with SARS-CoV-1. New England Journal of Medicine **382**(16), 1564–1567 (2020)

[13] Transport for London: S13171 A7: Station capacity planning. Mayor of London (2019)

[14] Agustina, L.G.: Improvement of voice alarm systems in underground stations. Proceedings of the Institute of Acoustics **35**(2), 1–16 (2013)

[15] Transport for London: Rolling Stock Information Sheets. Technical Report Information sheet: 4th edition (2014)

[16] United States Environmental Protection Agency: Exposure Factors Handbook: 2011 Edition vol. EPA/600/R-09/052F. National Center for Environmental Assessment, Washington, DC (2011)

[17] UK Health Security Agency: Vaccinations in London. Accessed at coronavirus.data.gov.uk (2022)
